# Up-regulation of lncRNA CASC9 promotes esophageal squamous cell carcinoma growth by negatively regulating PDCD4 expression through EZH2

**DOI:** 10.1186/s12943-017-0715-7

**Published:** 2017-08-30

**Authors:** Yuanyuan Wu, Liwen Hu, Yan Liang, Juan Li, Kai Wang, Xuedan Chen, Hui Meng, Xingying Guan, Kang Yang, Yun Bai

**Affiliations:** 1 Department of Medical Genetics, College of Basic Medical Science, Third Military Medical University, Chongqing, China; 2 Department of Cardiothoracic Surgery, Jinling Hospital, Medical school of Nanjing University, Nanjing, Jiangsu China; 3 Department of Cardiothoracic Surgery, Southwest Hospital, Third Military Medical University, Chongqing, China; 4 Department of clinical laboratory, Wuhan General Hospital of PLA, Wuhan, Hubei China

**Keywords:** lncRNA, ESCC, CASC9, PDCD4, And EZH2

## Abstract

**Background:**

Abnormal expression of numerous long non-coding RNAs (lncRNAs) has been reported in esophageal squamous cell carcinoma (ESCC) recently, but the great majority of their roles and mechanisms remain largely unclear. We aim to identify the critical ESCC-associated lncRNAs and elucidate the functions and mechanisms in detail.

**Methods:**

Microarrays were used to analyze the differentially expressed lncRNAs in ESCC tissues. qRT-PCR was used to verify the result of microarrays. The effects of the most up-regulated lncRNA, cancer susceptibility candidate 9(CASC9), on cell growth, proliferation and cell cycle were investigated by *in vivo* and *in vitro* assays. Microarrays and recovery tests were used to discover the regulatory targets of CASC9. RNA FISH and subcellular fractionation assays were used to detect the subcellular location of CASC9. Finally, the mechanism of CASC9 regulating PDCD4 was explored by RIP, RNA-protein pull down and ChIP assays.

**Results:**

ESCC tissue microarrays showed that CASC9 was the most up-regulated lncRNA. qRT-PCR analysis indicated that CASC9 expression was positively associated with tumor size and TNM stage, and predicted poor overall survival of ESCC patients. Knockdown of CASC9 inhibited ESCC cell growth *in vitro* and tumorigenesis in nude mice. Furthermore interfering CASC9 decreased cell proliferation and blocked cell cycle G1/S transition. CASC9-associated microarrays indicated that PDCD4 might be the target of CASC9. Consistent with this, PDCD4 expression was negatively associated with CASC9 expression in ESCC tissues and predicted good prognosis. Manipulating CASC9 expression in ESCC cells altered both PDCD4 mRNA and protein levels and cell cycle arrest caused by CASC9 knockdown could be rescued by suppressing PDCD4 expression. CASC9 located both in the nucleus and cytoplasm. Mechanistically, enhancer of zeste homolog2 (EZH2) could bind to both CASC9 and PDCD4 promoter region. Interfering CASC9 reduced the enrichment of EZH2 and H3K27me3 in the PDCD4 promoter region.

**Conclusions:**

Our study firstly demonstrates that lncRNA CASC9 functions as an oncogene by negatively regulating PDCD4 expression through recruiting EZH2 and subsequently altering H3K27me3 level. Our study implicates lncRNA CASC9 as a valuable biomarker for ESCC diagnosis and prognosis.

**Electronic supplementary material:**

The online version of this article (10.1186/s12943-017-0715-7) contains supplementary material, which is available to authorized users.

## Background

Esophageal cancer is one of the most commonly-diagnosed cancer type and ranks as the 6th most lethal cancer worldwide [1]. China has the highest incidence rate and esophageal squamous cell carcinoma (ESCC) is the predominant form [2, 3]. Because of lack of specific symptoms and effective early diagnostic methods, esophageal cancer tends to be diagnosed at a late stage and only 15%–25% of ESCC patients survive for 5 years after diagnosis [3]. Therefore, better understanding the molecular mechanisms of ESCC tumorigenesis and screening biomarkers are of significant importance for the improvement of early diagnosis and treatment of ESCC.

Long noncoding RNAs (lncRNAs) have been reported to drive many important cancer phenotypes by multiple ways [4], containing epigenetic modification, transcription regulation, RNA decay, miRNA sponging and so on [5, 6]. LncRNA expression is frequently dysregulated in various cancers and can predict prognosis [7–10]. The importance of lncRNAs in ESCC carcinogenesis is gradually coming to light recently. Several groups have reported the aberrant lncRNA expression profile in ESCC and identified hundreds of ESCC-associated lncRNAs [11–15], some of which could be used as biomarkers for diagnosis or prognosis of ESCC. For example, Li et al. has studied the lncRNA expression profile of ESCC by microarray including 119 pairs of tumor and normal tissues and found that a three-lncRNA signature (including the lncRNAs ENST00000435885.1, XLOC_013014 and ENST00000547963.1) is significantly associated with the overall survival of ESCC patients [15]. Tong and his colleagues have identified lncRNA POU3F3 could help to improve the efficiency of early ESCC screening [16].

However, compared with the accumulating number of ESCC-associated lncRNAs, only very few of them have been well-studied and have clear functions and mechanisms so far. These include linc-POU3F3, which is encoded by a genomic region next to POU3F3 and contributes to ESCC proliferation by reducing POU3F3 mRNA via enhancer of zeste homolog 2 (EZH2) [17] and lncRNA-uc002yug.2, which involves in the alternative splicing of RUNX1 by promoting the combination of RUNX1 and alternative splicing factors [18]. Our previous work shows that MALAT1 promotes proliferation and metastasis in ESCC by modifying the ATM-CHK2 pathway [19]. Therefore, the great majority of ESCC-associated lncRNAs need to be investigated in further detail.

In this study, we performed genome-wide lncRNA expression profile screening in 5 pairs of esophageal cancer and normal tissues by microarray to identify the novel promising cancer-related lncRNA in ESCC. Cancer susceptibility candidate 9 (CASC9, Gene ID 101805492 in NCBI records), which is the most up-regulated lncRNA in ESCC (GSE89102,fold change = 355.82385, *p* = 3.52E-06), predicts poor prognosis of ESCC. Following studies reveal its contribution to ESCC growth in vitro and in vivo, which is verified to occur in a PDCD4-dependent manner. Moreover, the mechanism of how CASC9 regulates PDCD4 expression is illustrated in detail.

## Methods

### Study population and tissue samples

All subjects in this study were genetically unrelated Chinese Han from Southwest China. The patients with ESCC underwent surgery resection and were pathologically diagnosed at Southwest Hospital, Third Military Medical University, China, during December 2006 to June 2014. A total of 91 ESCC tissues and 87 paired normal esophageal tissues were collected and stored at liquid nitrogen until use. The clinical characteristics of all patients were listed in (Additional file 1: Table S1). TNM staging of ESCC patients was according to the American Joint Committee on Cancer (AJCC). This study was approved by the ethics committee of the Third Military Medical University and informed consents were obtained before any operation to patients.

### Cell culture

Human esophageal cancer cell lines KYSE450, KYSE150, EC109 and EC9706 were purchased from Cell Bank of Chinese Academy of Sciences (Shanghai, China). Human normal esophagus epithelial cell line Het-1A was purchased from American Type Culture Collection (ATCC). The other cancer cells used in this study were kindly provided by colleagues in other departments. All cancer cells were cultured in RPMI-1640 medium (Hyclone, USA) containing 10% of fetal bovine serum, at 37 °C in a 5% CO2 cell culture incubator. KYSE450 and KYSE150 were genotyped for identity by STR method at Key Laboratory of Birth Defects and Reproductive Health (Chongqing, China) (Additional file 2: Fig. S1).

### Microarray screening and bioinformatic analysis

Five pairs of esophageal and adjacent non-tumor tissues used in the microarray screening were obtained from male patients and pathologically confirmed (Additional file 2: Fig. S2). The detailed information was provided in (Additional file 1: Table S2)***.*** RNA extraction and microarray hybridization were performed by Kangchen Company (Shanghai, China) using the Human lncRNA microarray v2.0 (8 × 60 K, arraystar, USA). Data were available via Gene Expression Omnibus (GEO) (GSE89102). Hierarchical clustering was performed using Cluster software to make salient the differential lncRNAs and mRNAs expression patterns. The Gene Co-expression Network between lncRNAs and mRNAs was analyzed by Cytoscape software. Gene Ontology (GO) analysis was performed to cluster the differentially expressed mRNAs (fold change >4) by defined terms or biological pathways.

Total RNA from the KYSE450 cells with CASC9 knockdown and control KYSE450 cells was isolated and quantified. The RNA integrity was assessed by standard denaturing agarose gel electrophoresis. The expression profiles were determined using the genechip Human Transcriptome Array 2.0 (Affymetrix, USA) by Qiming Bio-tech Company (Shanghai, China). Differentially expressed mRNAs (fold change >1.5) was clustered by GO analysis.

### RNA extraction and qRT-PCR

Total RNA from cells or tissues was extracted using Trizol reagent according to instructions (Takara, Japan), and then was reverse-transcribed into complementary DNA (cDNA). qRT-PCR was performed using SYBR Premix Ex Taq (Takara) on Illumina Eco Real-Time PCR System and Bio-Rad CFX Connect Real-Time PCR Detection System. Primers used in this study were listed in (Additional file 1: Table S3). The housekeeping gene glyceraldehyde-3-phosphate dehydrogenase (GAPDH) was used as internal control.

### RNA knockdown by small interfering RNAs

Small interfering RNAs (siRNAs) respectively targeting different sites of CASC9, EZH2 and PDCD4 and scrambled oligonucleotides used as negative control were designed and synthesized by Gene Pharma Company (Shanghai, China). The sequences of siRNAs and negative control were listed in (Additional file 1: Table S3).

For RNA interfering, cells were seeded on six-well plates at a density of 3 × 10^5^/well overnight, and then transfected with siRNA or negative control at a final concentration of 100 nM using Lipofectamine 2000 (Invitrogen, USA). RNA was extracted and the interfering efficiency was determined by qRT-PCR 48 h later.

### Cell viability, proliferation, apoptosis, and cell cycle assays

Cell viability was measured with Cell Counting Kit-8 (Dojindo Laboratory, Japan) every 24 h. Cell proliferation ability was detected by the Cell-light™ EdU Apollo® 567 In Vitro Imaging Kit (Ribobio, Guangzhou, China) 48 h later. Cell cycle distribution was analyzed by flow cytometry (BD Biosciences, USA) using propidium iodide (PI) staining (Beyotime, Shanghai, China) after 48 h–transfection and overnight fixation.

### Lentiviral constructs and xenografts in mice

Lentiviral vectors for SI2-CASC9 and NC were separately constructed by Gene Pharm company, and the stably transfected KYSE450 cell lines were established according to the manual. Two groups of four-week athymic female BALB/c mice were raised under specific pathogen-free conditions. A total of 1 × 10^7^ CASC9 stable knockdown cells or control cells were subcutaneously injected into a single side of the armpit of each mouse (*n* = 5 for each group). After 21-day injection, the mice were sacrificed by cervical dislocation without suffering, and the tumors were isolated and weighed. The experimental protocols were approved by the committee on animal experimentation of the Third Military Medical University.

### RNA–fish

CASC9 and U6 probes were synthesized by Bersinbio Company (Guangzhou, China). The slides of KYSE450 and KYSE150 cells were fixed in 4% paraformaldehyde for 20 min, and digested with protein K at 37 °C for 10 min. Then the slides were washed with PBS twice and dehydrated by ascending series of ethanol. After denatured at 73 °C for 3 min, 20 μL hybridization reaction solution (2 μL probes + 18 μL hybridization reaction) were added to the slides. The slides were hybridized at 42 °C overnight. After that, the slides were washed with 25% formamide /2 × Saline Sodium Citrate (SSC) at 53 °C twice and descending series of SSC at 42 °C. Finally, the slides were stained with DAPI and subjected to fluorescent signal detection using Zeiss LSM800 confocal laser microscopy (Zeiss, Germany).

ESCC tissues were fixed in 4% paraformaldehyde immediately after operation. After 72 h, the tissues were dehydrated in graded ethanol, cleared in dimethylbenzene and embedded in paraffin. After dewax and hydration, the paraffin sections were subjected to FISH as the cell slides.

### Subcellular fractionation

To determine the cellular localization of CASC9, nuclear fraction was isolated from cytoplasm according to the manufacturer’s instructions for NUCLEI EZ PREP NUCLEI ISOLATION KIT (Sigma, USA). Firstly, the cells were washed gently with ice-cold PBS twice. Then 1 ml ice cold Lysis Solution was added to the the 25cm^2^ flask and about 3 × 10^6^cells were harvested with a cell scraper on ice. The cell lysate was incubated on ice for 5 min. After centrifuging at 500 g for 5 min at 4 °C, the precipitate containing the nuclear RNA was isolated from the supernatant containing the cytoplasmic RNA. Finally, the supernatant was carefully removed to a new 1.5 ml EP tube. The precipitate was washed by PBS twice and resuspended by Nuclei EZ storage buffer.

### RNA-protein pull down assay

Full length of CASC9 and antisense-CASC9 was in vitro transcribed using TranscriptAid T7 High Yield Transcription Kit (ThermoFisher Scientific, USA) and labeled using Pierce RNA3’ End Desthiobiotinylation Kit (ThermoFisher Scientific). RNA pull down assay was performed using Pierce Magnetic RNA-Protein Pull down Kit (Thermo Fisher Scientific). 50 pmol desthiobiotinylation-labeled RNAs were mixed with 50 μl magnetic beads and then incubated with 200 μg protein lysate from KYSE150 or KYSE450 for 60 min at 4 °Cwith rotation. After washed 5 times with washing buffer, the RNA-binding proteins were eluted by 50 μl elution buffer and analyzed by Western Blotting.

### RNA immunoprecipitation

RNA immunoprecipitation (RIP) was performed using the EZ-Magna RIP RNA-Binding Protein Immunoprecipitation kit (Millipore, Germany) according to the manufacture’s instruction. The cells were washed with ice-cold PBS twice and harvested by a scraper. Then the cells were precipitated by centrifugation at 1500 rpm for 5 min at 4 °C and resuspended in 210 μl RIP Lysis buffer. The lysate was incubated on ice for 5 min. 5 μg EZH2 antibodies (Abcam, USA) or corresponding immunoglobulin G (IgG) was added to 50 μl magnetic beads and incubated with rotation for 30 min at room temperature. After that, 100 μl lysate was added to each tube and all the tubes were incubated with rotation overnight at 4 °C. The left 10 μl was used as Input. The RNA immunoprecipitation fraction was purified and detected by qRT-PCR. Primers are listed in (Additional file 1: Table S3).

### Chromatin immunoprecipitation assay

Chromatin immunoprecipitation (ChIP) was performed using the SimpleChIP® Enzymatic Chromatin IP Kit (CST, USA) according to the manufacturers’ instructions. Briefly, crosslinked chromatin was broken into 200to 1000 bp fragments by enzymatic digestion. The chromatin was immunoprecipitated using 2 μg anti-Ezh2 (CST), 2 μg anti-H3K27me3 (Millipore) and 2 μg corresponding IgG with rotation overnight at 4 °C. Then 30 μl magnetic beads were added to each tube and incubated with rotation for 2 h at 4 °C. The immunoprecipitated chromatin was washed with low salt solution three times and high salt solution once. Finally, the immunoprecipitated chromatin was purified and analyzed by qRT-PCR. Primers are listed in (Additional file 1: Table S3).

### Western blotting

Western blotting was performed as described previously [19]. The antibodies used were specific for EZH2 (1:1000, CST), PDCD4 (1:4000, CST), CCNE2 (1:1000, CST) and CDK6 (1:1000, CST). ECL chromogenic substrate was used to visualize the protein bands. GAPDH antibody (1:4000, KangChen, China) was used as the control.

### Statistical analysis

All statistical analyses were performed on SPSS 16.0. Measurement data are presented as mean ± standard deviation (SD). Statistical analyses were performed with Student’s t-test (two-tailed) and one-way ANOVA as appropriate. Kaplan-Meier Survival Analysis was used to evaluate cumulative survival probability. Correlation between CASC9 expression and PDCD4 mRNA expression in ESCC tissues was examined with Pearson correlation analysis. *P* < 0.05 was considered as significant.

## Results

### lncRNA expression profile in ESCC

**Fig. 1:**
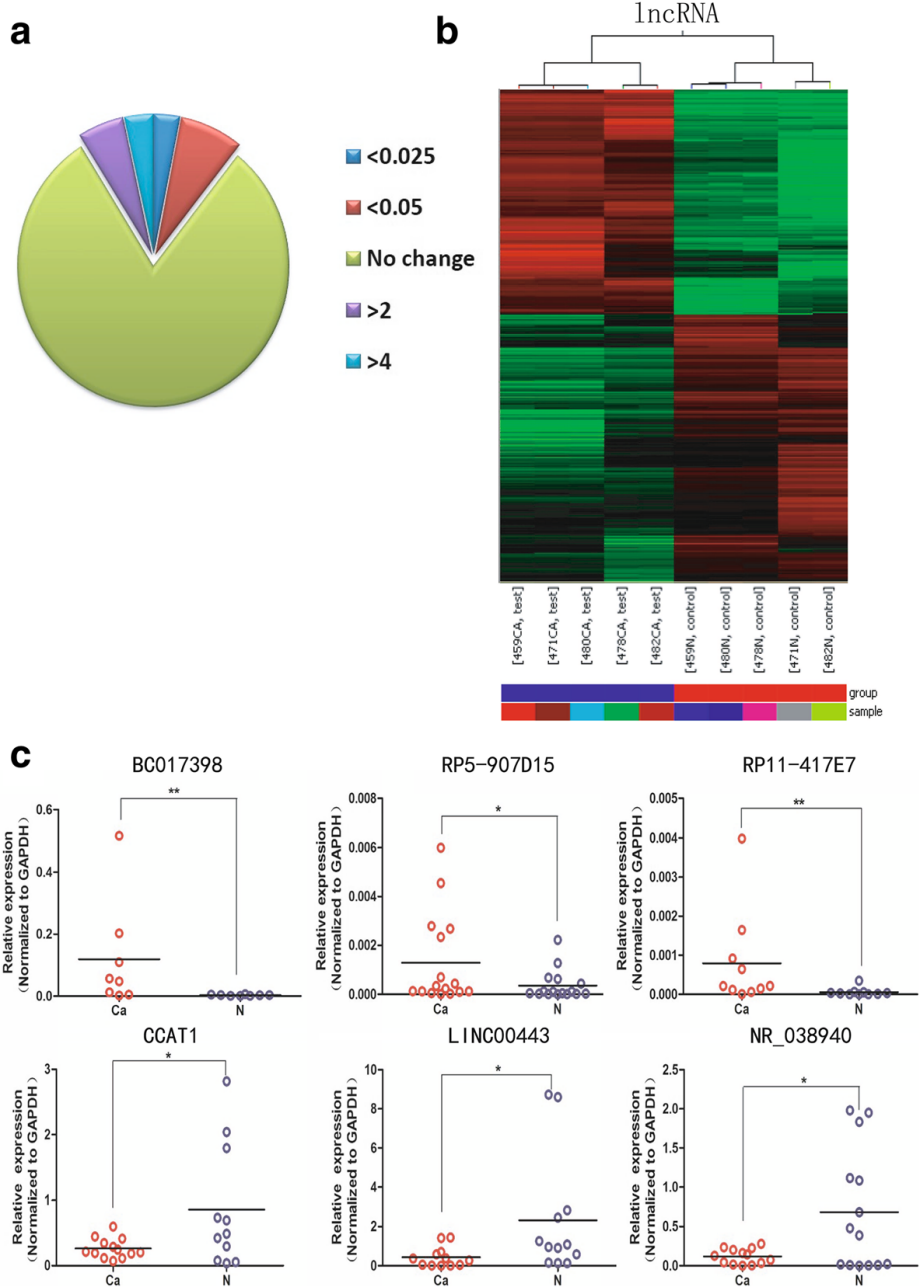
An overview of deregulated lncRNAs in ESCC specimens. **a** The proportion of deregulated lncRNAs in ESCC specimens. **b** Differentially expressed lncRNAs in ESCC and adjacent normal tissues. The lncRNA profiles from five paired ESCC tissues were clustered using Cluster 3.0. The red lines represented the up-regulated lncRNAs while the blue lines represented the down-regulated lncRNAs (*P* < 0.05 and fold change > 2). **c** The scatter plots showed the expression of selected lncRNAs in cancer (circle in red) and normal (circle inblue) tissues. *P < 0.05, **P < 0.01, ***P < 0.001

To identify the lncRNAs involved in ESCC progression, we selected 5 pairs of ESCC and adjacent normal tissues to conduct lncRNAs expression profiling, which included 24,011 probes in total. Microarray analysis revealed that 2939 (12.24%) lncRNA transcripts were consistently up-regulated (fold change > 2, *p* < 0.05), while 3517 (14.64%) ones were stably down-regulated (fold change < 0.5, *p* < 0.05) in ESCC tissues (Fig. 1a). These differentially regulated lncRNAs were clustered together to obviously distinguish ESCC samples from the normal ones (Fig. 1b).

Further analysis revealed that the deregulated lncRNAs in ESCC included many well-known tumor-associated lncRNAs, such as H19, TUG1 and SOX2OT, which have been reported to contribute to tumorigenicity in other cancers [20–22]. We randomly selected 3 up-regulated lncRNAs (BC017398, RP5-907D15, RP11-417E7) and 3 down-regulated lncRNAs (CCAT1, LINC00443, NR_038940) to detect their expression in ESCC and normal tissues by qRT-PCR. The results coincided with the microarray results well, confirming the reliability of our microarray results (Fig. 1c).

Additionally, we analyzed the differentially expressed mRNAs in ESCC tissues and conducted pathway analysis. Our results showed that the major alterations existed in cellular growth and proliferation function (Additional file 3), which underlay the etiology of ESCC. The Gene Co-expression Network between lncRNAs and mRNAs in ESCC tissues revealed that the expression of mRNAs was closely related to the expression of lncRNAs (Additional file 4). This finding indicated that the expression of mRNAs might be regulated by lncRNAs, which implied the prominent function of lncRNAs in ESCC carcinogenesis.

### CASC9 up-regulation predicts poor survival of ESCC patients

**Fig. 2:**
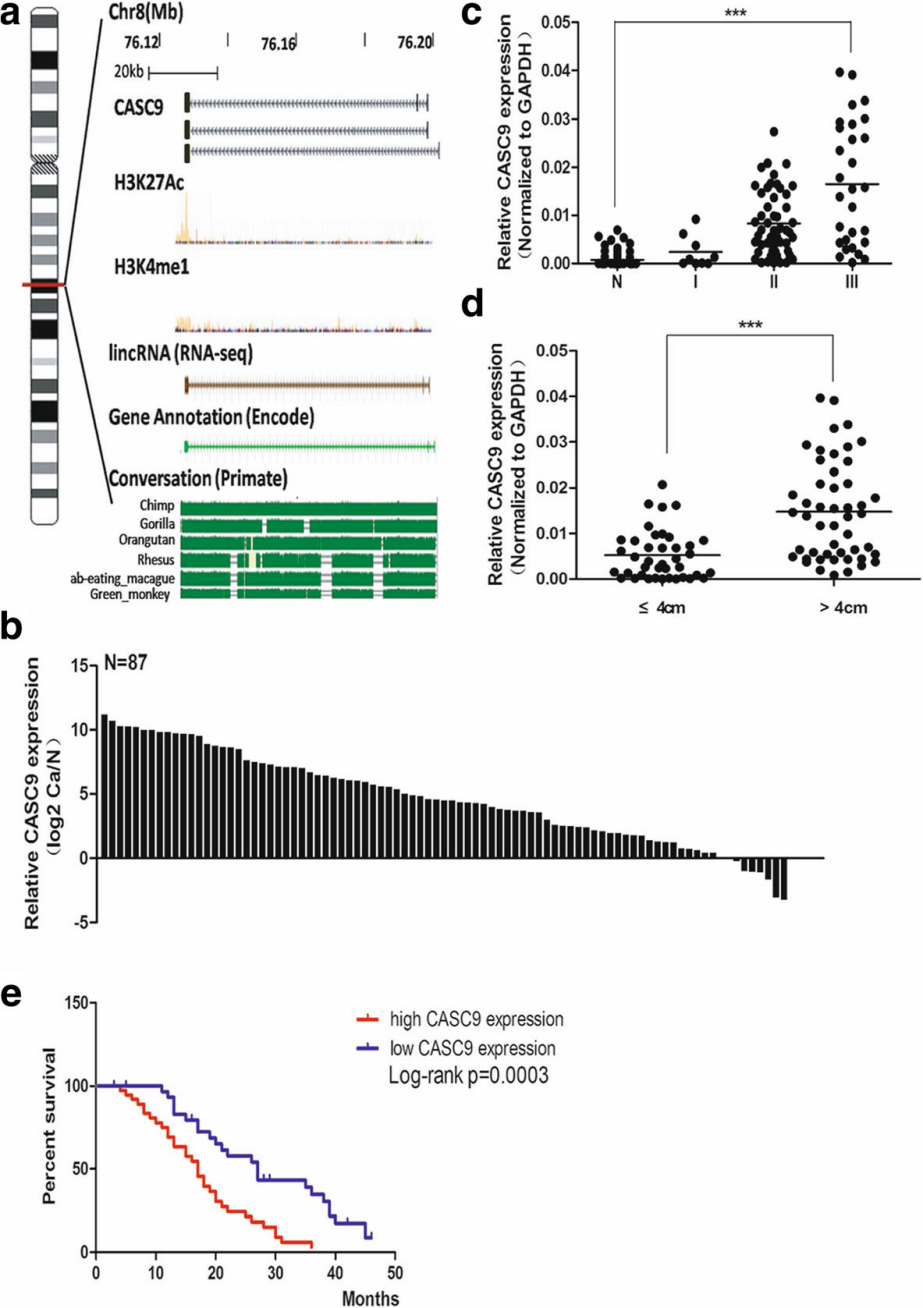
The architecture of CASC9 and its expression in ESCC specimens. **a** The genome architecture of CASC9 coding region. **b** qPCR analysis of CASC9 expression in 87 ESCC tissues and paired normal tissues. The results were expressed as log2 (2^-∆∆Ct^). Overexpression of CASC9 was detected in 90.8% (79/87) of ESCC tissues. **c** and **d** qPCR analysis of CASC9 expression in 91 ESCC tissues at different pathological stages and 87 paired normal tissues. CASC9 expression was significantly higher in patients at advanced pathological stages; one-way ANOVA analysis was performed to calculate the statistical difference. CASC9 expression was significantly higher in samples with larger tumor size. The statistical differences between the two groups were analyzed using unpaired Student’s t-test. **e** Survival analysis based on CASC9 expression levels in 69 cases of ESCC patients. The median level of CASC9 was used as cutoff. ESCC patients were divided into CASC9 high expression group and low expression group. The survival time of ESCC patients was compared between groups using the Log-Rank test, which indicated poor prognosis of patients in the high CASC9 expression group (*P* < 0.01). *** *P* < 0.001

Among all the differentially expressed lncRNAs, CASC9 was the most up-regulated one in ESCC. Therefore we focused on the gene architecture and expression of CASC9 to test whether it was possible to function in ESCC development or not. Bioinformatic analysis showed that CASC9 was located in a gene desert on chromosome 8q21.11. It had three transcripts. Almost no H3K27Ac and H3K4me1 histones modification signals were found in the CASC9 genome according to UCSC prediction, which indicated CASC9 was not an enhancer-like RNA. Taken together, CASC9 belonged to the intergenic lncRNA (lincRNA). The spanning chromosome region of CASC9 was highly conserved among primates, which indicated the evolutionary importance of CASC9 (Fig. 2a).

qRT-PCR was used to detect CASC9 expression (three transcripts) in 91 ESCC and 87 paired normal specimens. As expected, expression of CASC9 was elevated in 90.8% (79/87) ESCC tissues (Fig. 2b). Furthermore, correlation analysis of CASC9 expression with clinical characteristics revealed that CASC9 expression was increasing as the ESCC stage advanced (Fig. 2c). The dynamic change of CASC9 expression in ESCC progression indicated that CASC9 played an oncogenic role in ESCC and was a biomarker of ESCC staging. In addition, we found that ESCC tissues with larger size exhibited higher CASC9 expression (Fig. 2d).

We next divided 91 ESCC tissues into two groups according to CASC9 expression level and compared ESCC tumor size and TNM stage. Expectedly, the high expression group correlated with larger tumor size and more advanced stage (Additional file 1: Table S1). Kaplan–Meier survival analysis of ESCC patients showed that cases with higher expression of CASC9 exhibited poorer overall survival (*p* < 0.05, Fig. 2e). These data suggested that CASC9 may serve as an indicator for ESCC diagnosis and prognosis.

### Knockdown of CASC9 inhibits cell growth in vitro and in vivo

**Fig. 3:**
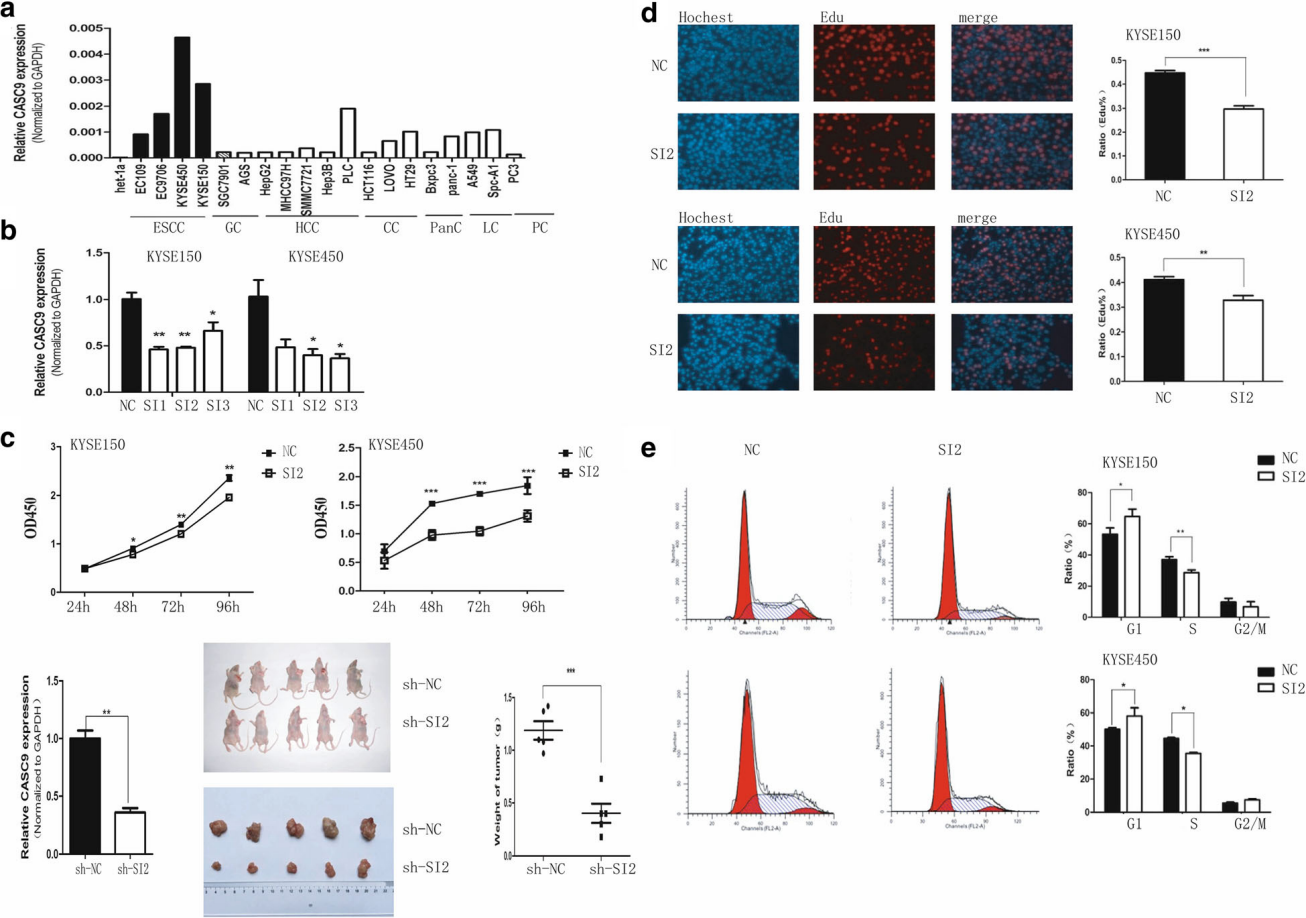
Knockdown of CASC9 inhibited esophageal cancer cells growth in vitro and in vivo. **a** Expression of CASC9 in normal esophageal epithelial cell line Het-1A, ESCC cell lines (EC109, EC9706, KYSE450, KYSE150) and other common cancer cell lines. GC, gastric cancer; HCC, hepatocellular carcinoma; CC, colonic cancer; PanC, pancreatic cancer; LC, lung cancer; PC, prostate cancer. CASC9 expression was significantly higher in ESCC cell lines compared to Het-1A and other common cancer cell lines. **b** Knockdown of CASC9 expression in KYSE150 and KYSE450 using small interfering RNA (si-RNA). SI2 was used in the further study for its stable efficiency. **c** Knockdown of CASC9 inhibited esophageal cancer cells growth in vitro and in vivo. CCK8 assay was performed to determine the cell growth of ESCC cells transfected with NC and SI2. Tumors developed from sh-CASC9 stably transfected KYSE450cells showed smaller volume and weights. **d** Edu assay was used to determine cell proliferation of KYSE150 and KYSE450 cells after transfected with si-RNA. The bar chart represented the ratio of Edu-positive cells. **e** Cell cycle was determined by flow cytometry analysis. The bar chart represented the percentage of cells in G0/G1, S, or G2/M phases respectively. Data were mean ± SD. *P < 0.05, **P < 0.01, ***P < 0.001

The biologic role of CASC9 in ESCC remains unclear. Therefore, we next performed loss-of-function studies to investigate its function in ESCC cells. First we detected the expression of CASC9 in some common cancer cells. A comparison of CASC9 expression in ESCC cell lines and other common cancers cells revealed that CASC9 was specifically up-regulated in ESCC cells (Fig. 3a). As CASC9 expression level associated with the ESCC tumor size significantly, we speculated that CASC9 might contribute to cell growth of ESCC.

Specific siRNAs targeting different sites of CASC9 transcript were employed to interfere CASC9 expression in KYSE450 and KYSE150, whose CASC9 expression were the highest in the detected ESCC cell lines. Both SI2-CASC9 and SI3-CASC9 effectively decreased CASC9 expression. The interfering efficiency of SI2-CASC9 was more stable in both cell lines, so it was chosen for further research (short for SI2 or SI-CASC9) (Fig. 3b). The CCK-8 assays showed that knockdown of CASC9 expression inhibited cell growth in both ESCC cell lines (Fig. 3c upper). In vivo experiments revealed that the tumors were much smaller and lighter in KYSE450-shCASC9 transplanted mice than those in control group (Fig. 3c down). Thus knockdown of CASC9 inhibited cell growth in vitro and in vivo.

Next, we investigated the effects of CASC9 knockdown on cell proliferation, apoptosis and cell cycle, which contributed to cell growth by different ways. 48 h after SI2-CASC9 transfection, proliferative cells labeled by Edu were significantly decreased (Fig. 3d). The cell cycle analysis demonstrated that inhibiting CASC9 expression led to cell cycle arrest in G1 phase and reduced the cell proportion in S phase (Fig. 3e), which was consistent with the result of Edu proliferation assays. Nevertheless, there was no statistical difference in apoptotic cells between CASC9 knockdown and control groups (Additional file 2: Fig. S3). These results suggested that interfering CASC9 might inhibit ESCC growth by reducing cell proliferating rate and blocking cell cycle rather than promoting cell apoptosis, indicating its possible involvement in cell cycle regulation.

### CASC9 promotes ESCC growth by negatively regulating PDCD4 expression

**Fig. 4:**
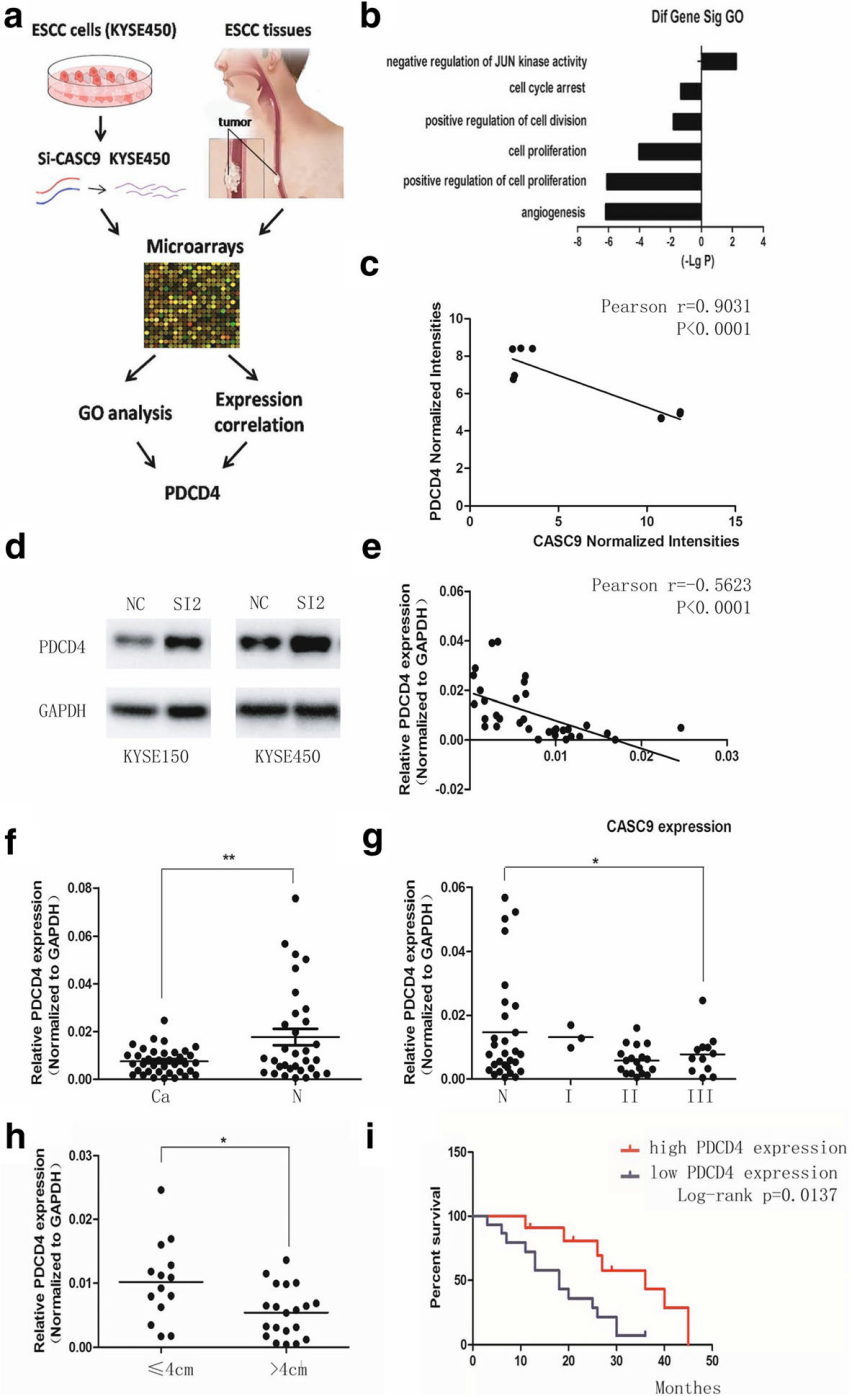
PDCD4 is a target gene of CASC9 and its expression correlates with clinical parameters in ESCC tissues. **a** Schematic flowchart showing the process of identifying PDCD4 as the target of CASC9. The combination of GO analysis and the gene expression correlation study identified PDCD4 in the cell growth pathways as potential regulatory target of CASC9. **b** Knockdown of CASC9 caused alterations in multiple pathways associated with cell growth. **c** Correlation analysis between CASC9 intensities and PDCD4 intensities provided by the microarray data from ESCC tissues. **d** Western blotting analysis showed that knockdown of CASC9 elevated the expression of PDCD4. **e** Spearman correlation was used to analyze the relationship between CASC9 and PDCD4 expression in another 37 ESCC tissues. **f**-**h** qPCR analysis of PDCD4 expression in ESCC tissues and adjacent normal tissues. PDCD4 expression was significantly lower in tumor tissues and especially lower in advanced pathological tumor samples. PDCD4 expression was higher in smaller tumor samples. Student’s t-test and one-way ANOVA analysis were performed to calculate the statistical difference. **i** Kaplan-Meier analysis showed that low PDCD4 expression indicated poor prognosis of ESCC patients. * *P* < 0.05, ** *P* < 0.01

To further unravel the mechanism how CASC9 promoted ESCC progression, we carried out microarray screening for CASC9-associated genes profile in KYSE450 cells. Being consistent with the CASC9 effect on cell growth, GO analysis of CASC9-associated genes showed significant alterations in cell proliferation and cell cycle pathways including negative regulation of JUN kinase activity, cell cycle arrest and so on (Fig. 4b), which conformed with the major alterations in ESCC tissues. Analyzed the correlation between CASC9 intensities and candidate genes intensities provided by the lncRNA and mRNA expression profile in ESCC tissues. Only VEGFC, PDCD4, PIK3CA, TMX1, PTHLH five genes expression was significantly associated with CASC9 expression as expected (Fig. 4c, Additional file 2: Fig. S4), which indicated that they might be the downstream of CASC9 in vivo.

In order to confirm the result, qRT-PCR was used to detect target genes expression after manipulating CASC9 expression in KYSE450. Knockdown of CASC9 decreased the expression of PIK3CA, VEGFC, TMX1, PTHLH and increased the expression of PDCD4 at mRNA level (Additional file 2: Fig. S5A, B), which was consistent with the microarray result. However, overexpression of CASC9 could only reduce PDCD4 mRNA expression and had no effect on the expression of other four genes (Additional file 2: Fig. S5C, D). This might owe to the poor efficiency of overexpression. This finding suggested that PDCD4 might be a direct target of CASC9, so it was more sensitive to the change of CASC9 expression than the other genes. Furthermore, knockdown of CASC9 elevated the PDCD expression at protein level in KYSE150 and KYSE450 (Fig. 4d).

Next, we detected PDCD4 expression in 37 pairs of ESCC tissues and analyzed the association between its expression and clinical characteristics. Strikingly, PDCD4 expression was in inversely correlated with CASC9 expression in ESCC tissues (Fig. 4e). Contrary to CASC9, PDCD4 was down-regulated in ESCC tissues (Fig. 4f). The expression of PDCD4 decreased as the ESCC stage advanced (Fig. 4g). ESCC tissues with smaller maximum diameter had a higher PDCD4 expression (Fig. 4h). High PDCD4 expression predicted good prognosis (Fig. 4i). These findings indicated that PDCD4 was a reliable target gene of CASC9 and CASC9 might promoted ESCC growth through negatively regulating PDCD4. To further unveil the relationship between CASC9 and PDCD4, we analyzed the correlations of their expression with the other lncRNAs identified in Fig. 1c. None of them correlated with CASC9 and PDCD4 at the same time (Additional file 2: Fig. S6), which indicated that CASC9 regulated PDCD4 in a specific way.

**Fig. 5:**
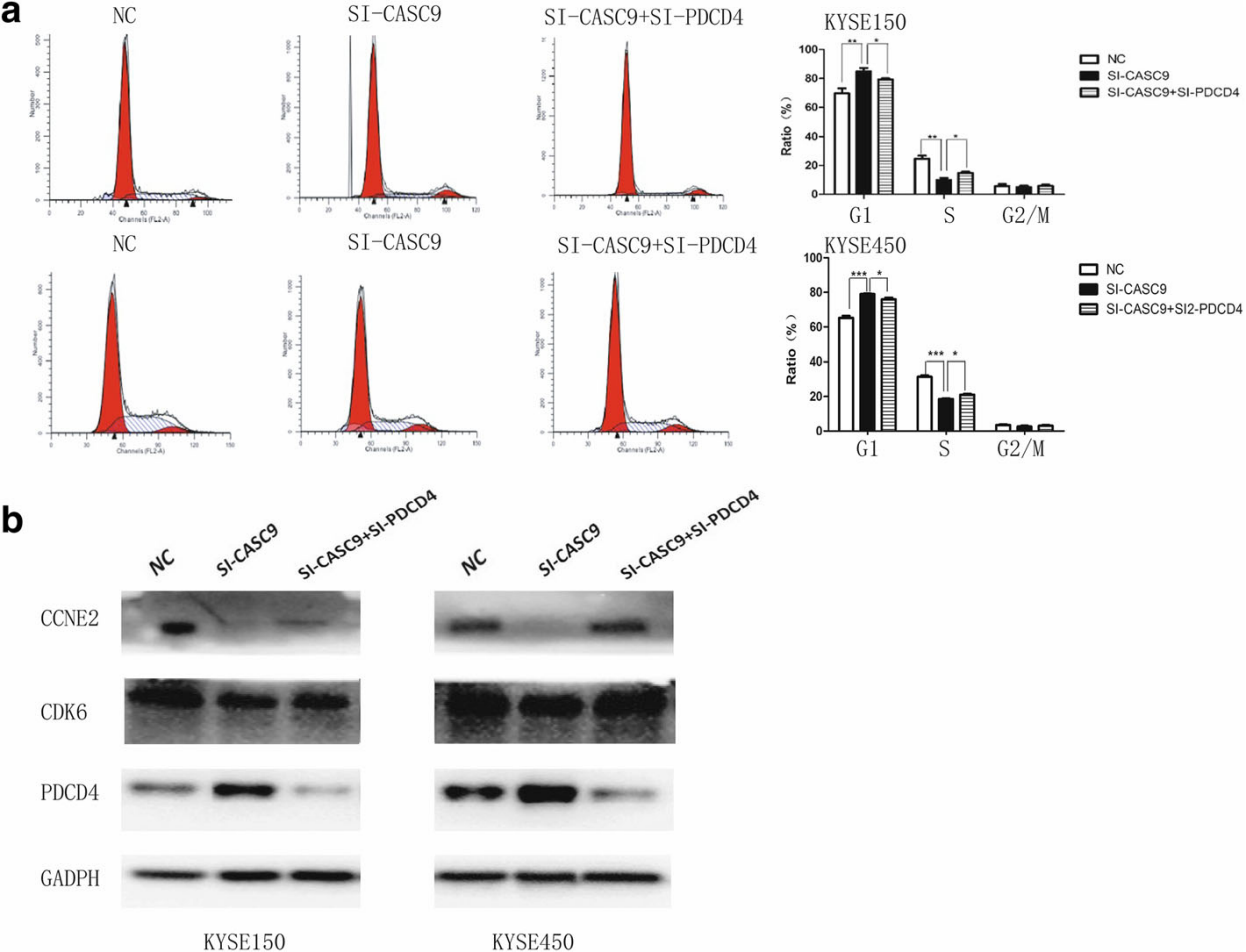
The effect of CASC9 interfering on cell cycle was partly neutralized by suppressing PDCD4 expression. **a** The bar chart represented the percentage of cells in G0/G1, S, or G2/M phases respectively. Data were mean ± SD. *P < 0.05, **P < 0.01, ***P < 0.001. **b** Western blotting analysis of molecular markers on G1/S phase in KYSE150 and KYSE450 cells. Knockdown of CASC9 caused down-regulation of CCNE2 and CDK6, while interfering PDCD4 rescued this effect

The tumor-suppressor role of PDCD4 has been characterized well, and PDCD4 knockdown could significantly promote cell growth [23, 24]. Thus it is reasonable to hypothesize that the suppressive effect of CASC9 knockdown on cell growth could be rescued by suppressing PDCD4. By cell cycle assays, we found that interfering PDCD4 partly rescued cell cycle G1/S arrest caused by CASC9 knockdown (Fig. 5a). Western blotting demonstrated that knockdown of CASC9 reduced the expression of CCNE2 and CDK6, biomarkers of S phase, while suppressing PDCD4 rescued its expression (Fig. 5b). These findings suggested that CASC9 might confer its effects on ESCC progression in a PDCD4-dependent manner.

### CASC9 regulates PDCD4 expression via EZH2

To investigate the potential mechanism of CASC9 regulating PDCD4 expression in ESCC, we first performed bioinformatic analysis and found that the promoter region of PDCD4 was enriched in the repressive mark H3K27me3 and EZH2 binding sites in various cells (Additional file 2: Fig. S7), implying PDCD4 expression under regulation of epigenetic modification. As approximately 20% lincRNAs can bind EZH2, we wondered whether CASC9 regulated PDCD4 expression through EZH2.

**Fig. 6:**
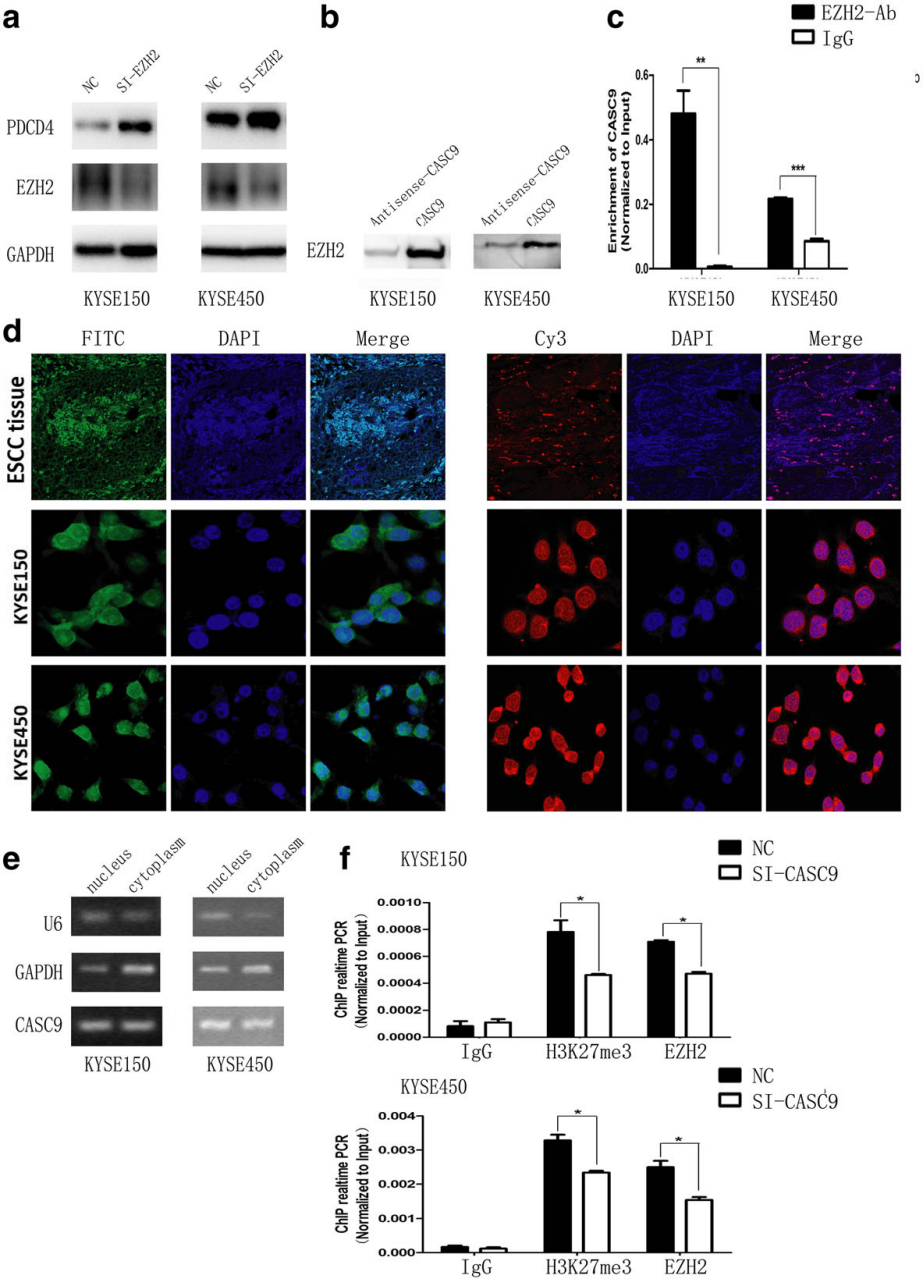
CASC9 regulated PDCD4 via EZH2. **a** Knockdown of EZH2 increased the expression of PDCD4, which was detected by Western blotting. **b** RNA-protein pull down indicated the binding of CASC9 with EZH2. **c** RIP showed that CASC9 was significantly enriched with EZH2 antibody compared with lgG in KYSE150 and KYDE450 cells. **d** CACS9 was distributed in both nucleus and cytoplasm analyzed by FISH CASC9 was labeled by FITC (green) and U6 was labeled by Cy3 (red). Nucleus was stained by DAPI (blue). The ESCC tissue sections were observed in 200X magnification to show a complete keratin pearl. The slides of ESCC cells were observed in 630X magnification. **e** CACS9 was distributed in both nucleus and cytoplasm analyzed by subcellular fractionation assay. **f** Knockdown of CASC9 decreased the enrichment of EZH2 and H3K27me3 in the PDCD4 promoter region in ESCC cells (KYSE150 and KYSE450). Data were mean ± SD. *P < 0.05, **P < 0.01,***P < 0.001

To prove this hypothesis, we first used ChIP assay to detect the binding of EZH2 and PDCD4 promoter region in ESCC cells without any treatment (Additional file 2: Fig. S8). The result demonstrated that EZH2 could bind PDCD4 promoter region. Then knockdown of EZH2 by siRNA promoted PDCD4 mRNA and protein expression in ESCC cell lines (Additional file 2: Fig. S9, Fig. 6a), supporting the idea that PDCD4 expression was regulated by EZH2.

After that, we tested whether CASC9 could bind EZH2 or not. As expected, CASC9 bound more EZH2 than antisense-CASC9 by RNA-protein pull down and Western blotting assays (Fig. 6b). This binding was validated by RIP experiment (Fig. 6c). Then we detected the subcellular location of CASC9. RNA-FISH results revealed that CASC9 was distributed both in the nucleus and in the cytoplasm of ESCC cells and tissue (Fig. 6d), which was verified by the nucleus-cytoplasm isolation assay (Fig. 6e). Finally we wondered whether CASC9 could affect the binding between EZH2 and PDCD4 promoter region. Using ChIP and qRT-PCR assays, we found that CASC9 knockdown decreased the enrichment of EZH2 and H3K27me3 in the PDCD4 promoter region (Fig. 6f), which resulted in up-regulating PDCD4 expression. Taken together, these results suggested that CASC9 negatively regulated PDCD4 expression by recruiting EZH2 to its promoter region and increasing the H3K27me3 enrichment of its promoter.

## Discussion

ESCC is one of the most fatal cancers worldwide, but the etiology remains poorly understood. To explore the pathogenesis of ESCC and find novel biomarker for ESCC, we conducted a microarray analysis to screen the whole transcripts expression profile in ESCC. Our results reveal the significant alterations in cell growth, indicating that cell growth is closely related to the malignant characteristics of ESCC. Intriguingly, we have identified an aberrantly overexpressed lncRNA CASC9 in ESCC tissues, which has an important role in cell growth.

Among all the ESCC lncRNA expression profile studies, lncRNA CASC9 has been also reported to be extremely up-regulated in ESCC tissues by Cao’s and Xu’s teams using another microarray platform and RNA sequencing technology [12, 13], which confirms the accuracy of our microarray result and implies the crucial role of CASC9 in the development of ESCC as well.

Following qRT-PCR results suggested that lncRNA CASC9 expression is elevated in most ESCC tissues, especially in advanced samples with larger tumor size, and its higher expression predicts a poor clinical outcome. Moreover, it has been reported that higher CASC9 expression correlates with the differentiation of ESCC [12]. It is worth noting that Xu and his colleagues didn’t observe a correlation of CASC9 expression with tumor size. Considering only 42 ESCC tissues collected in their study (less than half of our study), we think that limited sample size is the major reason why they failed to find this correlation. For the first time, we describe the close relationship between CASC9 expression and clinical characteristics of ESCC in detail. Compared with other cancer cells, CASC9 is specifically highly expressed in ESCC cells, which indicates that the expression of CASC9 is relatively tissues-specific. All these above make lncRNA CASC9 a potential and significant biomarker for ESCC diagnosis and prognosis.

As Cao and Xu respectively just did simple experiments in vitro to indicate that CASC9 may promote ESCC growth and metastasis, we took in vivo and in vitro assays to further characterize the role of CASC9 in ESCC. Knockdown of CASC9 inhibited ESCC growth in vivo and in vitro. Subsequent investigations showed that interfering CASC9 reduced cell proliferation and induced cell cycle arrest at G1/S phase. But it made no difference to apoptosis in KYSE150 and KYSE450. These findings confirm the functional role of CASC9 on cell growth.

To our knowledge, there is no article reporting the mechanism of CASC9. Different from other ESCC-associated lncRNAs, such as POU3F3 and uc002yug.2, regulating their nearby coding genes [17, 18], CASC9 is located in a gene desert without any genes nearby. So it is a challenge for us to study the mechanism of CASC9. Mechanisms of lncRNAs regulating genes expression may be partially dependent on their subcellular location [25]. So, we firstly performed microarray to analyze the mRNAs whose expression were changed after altering CASC9 levels and detected the subcellular location of CASC9. Then, functional screening and recovery experiments were used to validate the potential targets. Subsequently, reasonable assumptions about the mechanism of CASC9 regulation was put forward according to the bioinformatic analysis of target genes and CASC9 subcellular location.

Microarray result revealed that pathways on cell proliferation and cell cycle were significantly changed, which was consistent with the function of CASC9. Among these affected-genes which related to cell growth, a well-characterized tumor suppressor gene PDCD4 was identified as a potential downstream target of CASC9. qRT-PCR analysis indicated that PDCD4 expression was negatively associated CASC9 expression in ESCC tissues. Knockdown of CASC9 could downregulate PDCD4, while overexpression of CASC9 increased PDCD4 levels. PDCD4 is reported to be involved in apoptosis, proliferation and cell cycle [26–28]. Our previous work has also revealed that suppression of PDCD4 promotes G1/S transition of ESCC cells [24], which is consistent with present results in CASC9 knockdown cells. In this study, we further observed that suppression of PDCD4 rescued the cell cycle arrest caused by CASC9 knockdown, further sustaining the viewpoint that CASC9 performs in a PDCD4-dependent manner. Göke et al. have found that overexpression of PDCD4 could reduce the activity of CDK4/6 and CDK2 by inducing p21^Waf1/Cip1^ [29]. CCND1-CDK4/6 and CCNE-CDK2 are responsible for progression of G1 to S phase [30–32]. We found that knockdown of CASC9 reduced the expression of CDK6, and CCNE2, and this could be dampened by knockdown of PDCD4. Thus, based on the above results, we considered that CASC9 promoted ESCC cell growth partially by downregulating PDCD4 expression.

Then we explored how CASC9 regulated PDCD4 expression. Bioinformatic analysis and EZH2 knockdown assay proved that PDCD4 is regulated by EZH2. As CASC9 negatively regulates PDCD4 expression and many classic lincRNAs can bind EZH2 [33, 34], we hypothesized that CASC9 could modify chromosome by recruiting EZH2 to chromosome regions of target genes. EZH2 is a subunit of polycomb repressive complex 2 (PRC2), which has an effect of H3K27me3 and leads to repressing gene expression. It was first reported that lncRNA HOTAIR silences the tumor suppressor genes by interacting with EZH2 and enhancing H3K27me3 [35]. We then confirmed the binding of CASC9 and EZH2 by RNA-protein pulldown assay and RIP experiment. In addition, we found that CASC9 is distributed both in the nucleus and cytoplasm, which corroborates the hypothesis from the location side. Finally, we verified that knockdown of CASC9 interfered the binding affinity of EZH2 to the promoter of PDCD4 and decreased the H3K27me3 level of its promoters. Taken together, these findings support the idea that CASC9 may suppress PDCD4 expression by epigenetic mechanism and CASC9 executes its oncogenic effects through a PDCD4-dependent way. It is a significant mechanism of nuclear localized CASC9. As for the cytoplasmic localized CASC9, it may regulate other downstream pathways by functioning as miRNA sponge or involving in the synthesis and chemical modification of proteins [36–38].

It is universal that lncRNAs regulate target genes by interacting with EZH2. We prove the significant importance of this mechanism in ESCC. As an important tumor suppressor, we know much about its involvement in carcinogenesis, but little about the upstream regulators of PDCD4. Previous studies have shown that microRNAs including miR-182 [39], miR-183 [40] and miR-21 [41] participate in the regulation of PDCD4. But the role of lncRNAs in PDCD4 regulation remains unclear. Here we report its expression regulated by lncRNAs and epigenetic mechanism for the first time.

## Conclusion

Our study, for the first time, reports that lncRNA CASC9 may serve as a prognostic biomarker of ESCC and reveals the mechanism of lncRNA CASC9 on regulating cell growth. LncRNA CASC9 promotes ESCC growth by negatively regulating PDCD4 via EZH2. As CASC9 has been reported to involve in metastasis and drug resistance [42–44], it has become to a promising oncogene of clinical value and more work is needed to uncover the mechanisms of CASC9’s oncogenic function.

## Additional files

Additional file 1: Table S1. Correlation between CASC9 expression and clinicopathological characteristics of ESCC patients. **Table S2.** General clinical characteristics of ESCC patients used for microarrays. **Table S3.** All primers and siRNA sequences used in this study (DOCX 17 kb)

Additional file 2: Fig. S1. The STR identification of KYSE150 and KYSE450. **Fig. S2.** HE staining of ESCC and adjacent tissues. **Fig. S3.** Cell apoptosis was determined by flow cytometry analysis. **Fig. S4.** Correlation analysis of CASC9 intensities and candidate genes intensities provided by the ESCC tissue profiles. **Fig. S5.** qRT-PCR was used to detect the mRNA expression of target genes after interfering and overexpressing CASC9. **Fig. S6.** Correlation between transcripts identified in Fig.1 and CASC9 or PDCD4. **Fig. S7.** ChIP-seq data from Encode indicate that the promoter region of PDCD4 is enriched in the H3K27me3 and EZH2 binding sites. **Fig. S8.** ChIP assay showed that EHZ2 could bind to the region of PDCD promoter. **Fig. S9.** PDCD4 mRNA expression after interfering EZH2 (DOCX 14348 kb)

Additional file 3: GO analysis of differentially expressed mRNAs (XLS 546 kb)

Additional file 4: The Gene Co-expression Network between lncRNAs and mRNAs in ESCC tissues (TIFF 8164 kb)
