# Supplementary material for: Up-regulation of lncRNA CASC9 promotes esophageal squamous cell carcinoma growth by negatively regulating PDCD4 expression through EZH2
Source: Mol Cancer. 2017 Aug 30;16:150. doi: 10.1186/s12943-017-0715-7 (PMC5577767; doi:10.1186/s12943-017-0715-7)
Supplement: Supplementary file 1 — Correlation between CASC9 expression and clinicopathological characteristics of ESCC patients. Table S2. General clinical characteristics of ESCC patients used for microarrays. Table S3. All primers and siRNA sequences used in this study (DOCX 17 kb) [file 12943_2017_715_MOESM1_ESM.docx]

Table S1. Correlation between CASC9 expression and clinicopathological characteristics of ESCC patients.

| Clinicopathological parameter |  | CASC9 expression | | P-Value^a^ |
| --- | --- | --- | --- | --- |
|  |  | High expression | Low expression |  |
| Age(year) | ≤55 | 17 | 19 | 0.7309 |
|  | >55 | 28 | 27 |  |
| Sex | Female | 10 | 13 | 0.5075 |
|  | Male | 35 | 33 |  |
| Smoking* | Never | 13 | 19 | 0.1864 |
|  | Ever | 32 | 26 |  |
| Drinking* | Never | 12 | 17 | 0.2594 |
|  | Ever | 33 | 28 |  |
| TNM stage* | I | 1 | 8 | ***0.0281*** |
|  | II | 26 | 25 |  |
|  | III | 18 | 11 |  |
| Maximum*  diameter | ≤4.0 cm | 14 | 26 | ***0.0080*** |
|  | >4.0cm | 31 | 18 |  |

a Chi-square test results;

*clinical information of some patients is not complete.

Table S2. General clinical characteristics of ESCC patients used for microarrays.

| Sample ID | Age (year) | Sex | Smoking | Drinking | TNM stage |
| --- | --- | --- | --- | --- | --- |
| 459 | 60 | male | ever | ever | T2N0M0 |
| 471 | 58 | male | ever | ever | T2N0M0 |
| 478 | 50 | male | ever | ever | T2N0M0 |
| 480 | 64 | male | ever | ever | T2N0M0 |
| 482 | 61 | male | ever | ever | T2N0M0 |

Table S3. All primers and siRNA sequences used in this study.

| Name | Sequence |
| --- | --- |
| **For qRT-PCR** |  |
| GAPDH-F | GGGAGCCAAAAGGGTCATCA |
| GAPDH-R | TGATGGCATGGACTGTGGTC |
| BC017398-F | GCCAACTAGGCTGCTGCTAA |
| BC017398-R | TTGTGCAGCAGTCACTCAAGA |
| RP5-907D15-F | CAAGGTCAGGCCATACCCAC |
| RP5-907D15-R | CAGGTTCTGTAGGGGAGCAC |
| RP11-417E7-F | CAACACTGAAACCGCCTGAC |
| RP11-417E7-R | AAGGAAGACAGACACGAGGC |
| CCAT1-F | GCCGTGTTAAGCATTGCGAA |
| CCAT1-R | TCATGTCTCGGCACCTTTCC |
| LINC00443-F | ATGCTCCGGAGGGCTTATTG |
| LINC00443-R | AGCGCATGGTGTCAACAGAT |
| NR_038940-F | CAGTGAAGCCCACGAACGTA |
| NR_038940-R | GTTCACGATGTGAGCCCAGT |
| CASC9-F | TTGGTCAGCCACATTCATGGT |
| CASC9-R | AGTGCCAATGACTCTCCAGC |
| PDCD4-F | AGTGACGCCCTTAGAAGTGG |
| PDCD4-R | TCATATCCACCTCCTCCACA |
| PIK3CA-F | CGTTTCTGCTTTGGGACAAC |
| PIK3CA-R | CCTGATGATGGTCGTGGAG |
| VEGFC-F | GCTTCTTCTCTGTGGCGTGT |
| VEGFC-R | ACACAGACCGTAACTGCTCC |
| TMX1-F | GCTGGAAGAGCTGAGTTGTA |
| TMX1-R | ATGGCAAGTCCTGATCCACA |
| PTHLH-F | TTCCTCCGCTCGGTTTTCAA |
| PTHLH-R | CCACTGCTGAACCAGTCTCC |
| EZH2-F | GGACTCAGAAGGCAGTGGAG |
| EZH2-R | CTTGAGCTGTCTCAGTCGCA |
|  |  |
| **For ChIP-qPCR** |  |
| PDCD4-F | GGTCTGGGAAGCTCCGATTT |
| PDCD4-R | GCAGTTGGTGGTCATCCTCA |
| NC(SOX2)-F | GCCCTGCAGTACAACTCCAT |
| NC(SOX2)-R | GACTTGACCACCGAACCCAT |
|  |  |
| **siRNAs** |  |
| NC | UUCUUCGAACGUGUCACGUTT |
| SI1-CASC9/SI1 | GGGCAUUGAGAAGUUAGAATT |
| SI2- CASC9/SI2/SI-CASC9 | GCCUGUGAUAGCAGAACAATT |
| SI3-CASC9/SI3 | GGACUCAUAUUACCAGUCUTT |
| PDCD4-SI | CAUUCAUACUCUGUGCUGG |
| SI1-EZH2 | GGAUGGUACUUUCAUUGAATT |
| SI2-EZH2/SI-EZH2 | CGGCUUCCCAAUAACAGUATT |
| SI3-EZH2 | GAGGGAAAGUGUAUGAUAATT |
