# Supplementary material for: Up-regulation of lncRNA CASC9 promotes esophageal squamous cell carcinoma growth by negatively regulating PDCD4 expression through EZH2
Source: Mol Cancer. 2017 Aug 30;16:150. doi: 10.1186/s12943-017-0715-7 (PMC5577767; doi:10.1186/s12943-017-0715-7)
Supplement: Supplementary file 2 — The STR identification of KYSE150 and KYSE450. Fig. S2. HE staining of ESCC and adjacent tissues. Fig. S3. Cell apoptosis was determined by flow cytometry analysis. Fig. S4. Correlation analysis of CASC9 intensities and candidate genes intensities provided by the ESCC tissue profiles. Fig. S5. qRT-PCR was used to detect the mRNA expression of target genes after interfering and overexpressing CASC9. Fig. S6. Correlation between transcripts identified in Fig.1 and CASC9 or PDCD4. Fig. S7. ChIP-seq data from Encode indicate that the promoter region of PDCD4 is enriched in the H3K27me3 and EZH2 binding sites. Fig. S8. ChIP assay showed that EHZ2 could bind to the region of PDCD promoter. Fig. S9. PDCD4 mRNA expression after interfering EZH2 (DOCX 14348 kb) [file 12943_2017_715_MOESM2_ESM.docx]

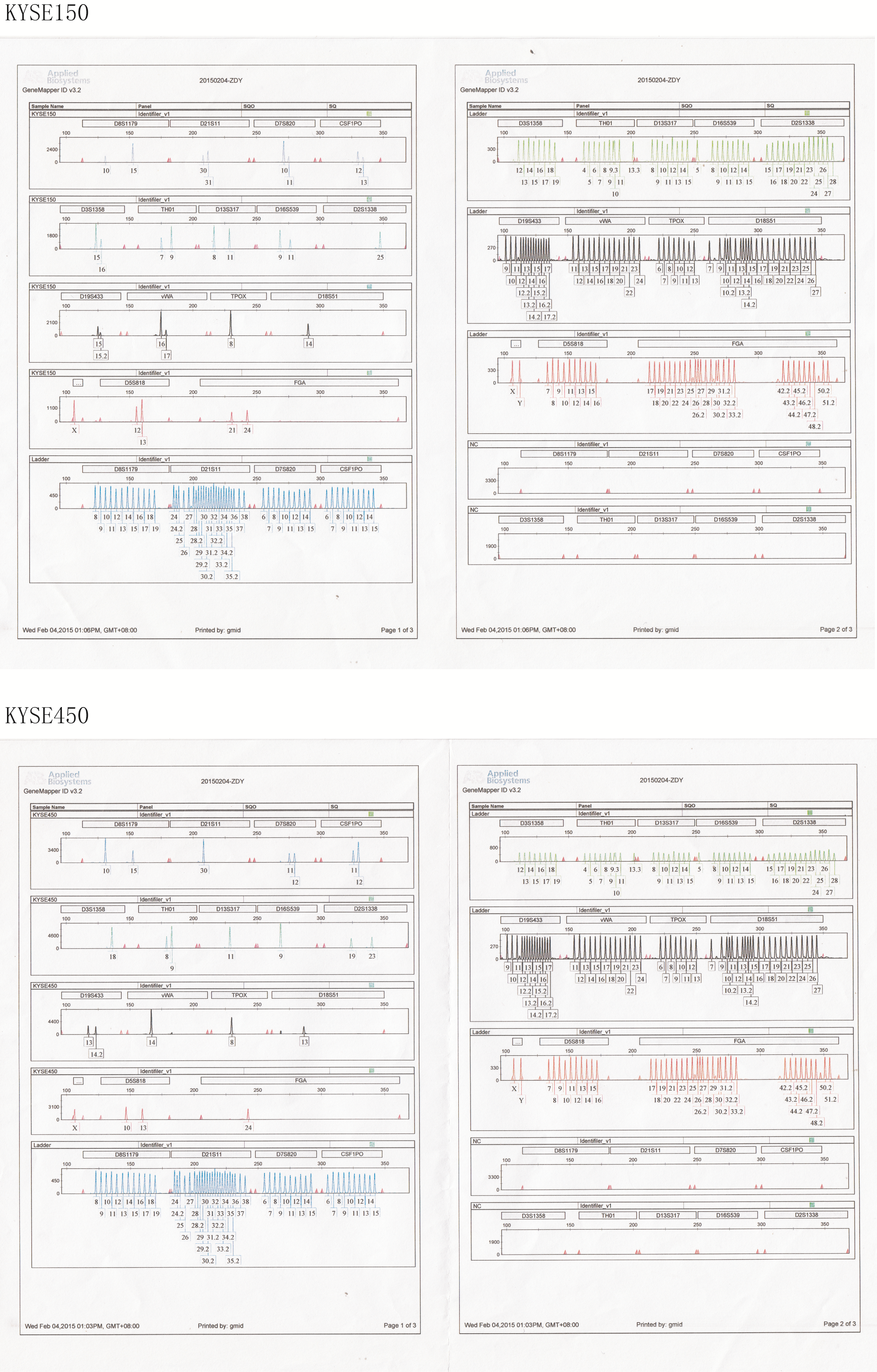


**FigureS1.** The STR identification of KYSE150 and KYSE450.


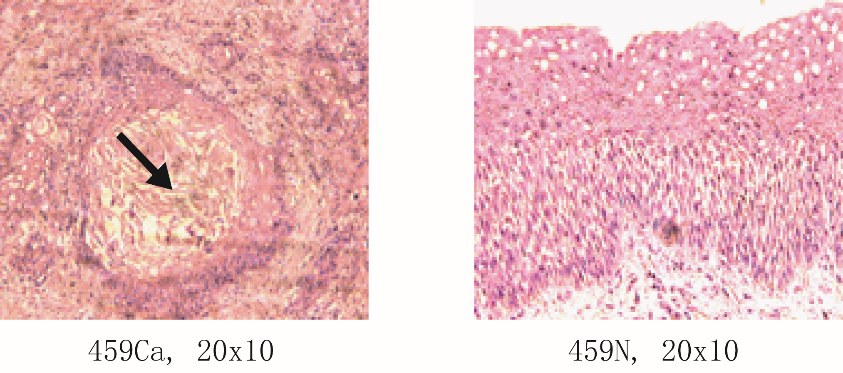


**FigureS2.** HE staining of ESCC and adjacent tissues. The arrow points to a keratin pearl which is regarded as the typical sigh of squamous cell carcinoma.


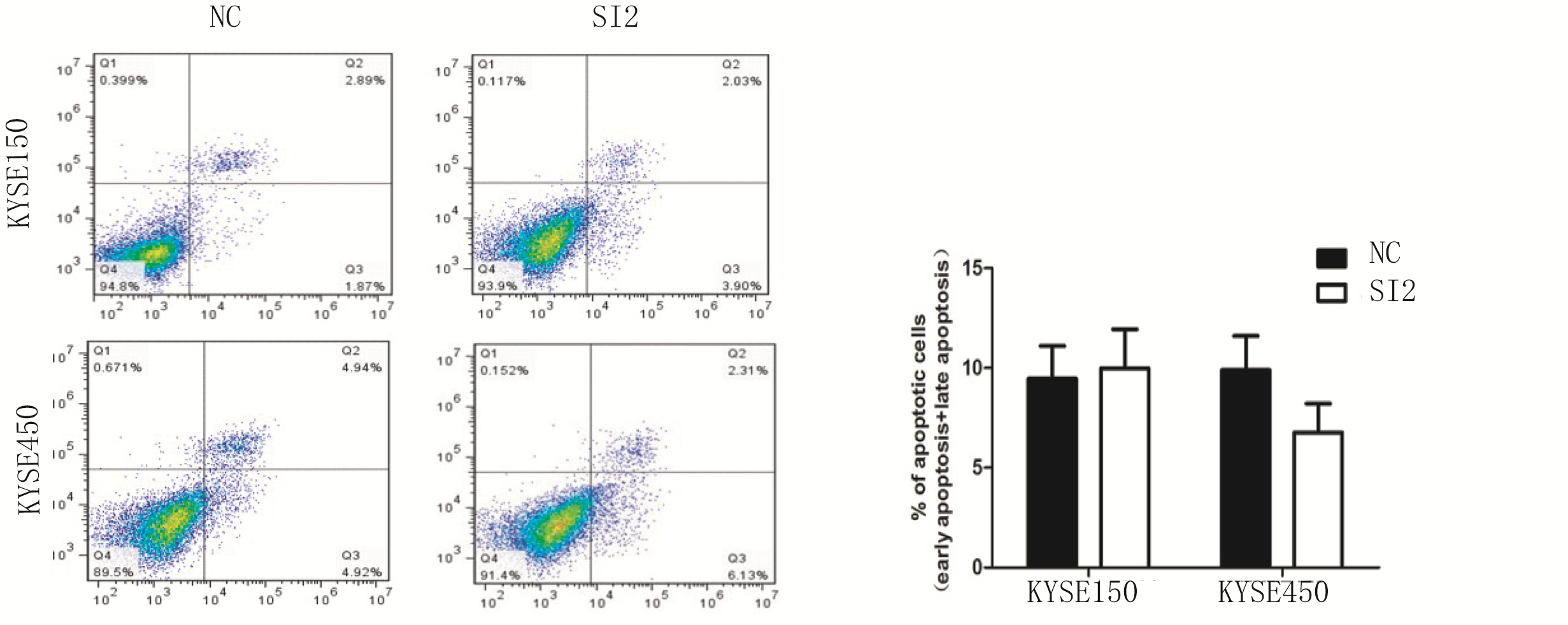


**FigureS3.** Cell apoptosis was determined by flow cytometry analysis. The bar chart represented the sum percentage of early and late apoptotic cells respectively. Data were mean ± SD. No significant difference was found between NC and SI2 groups.


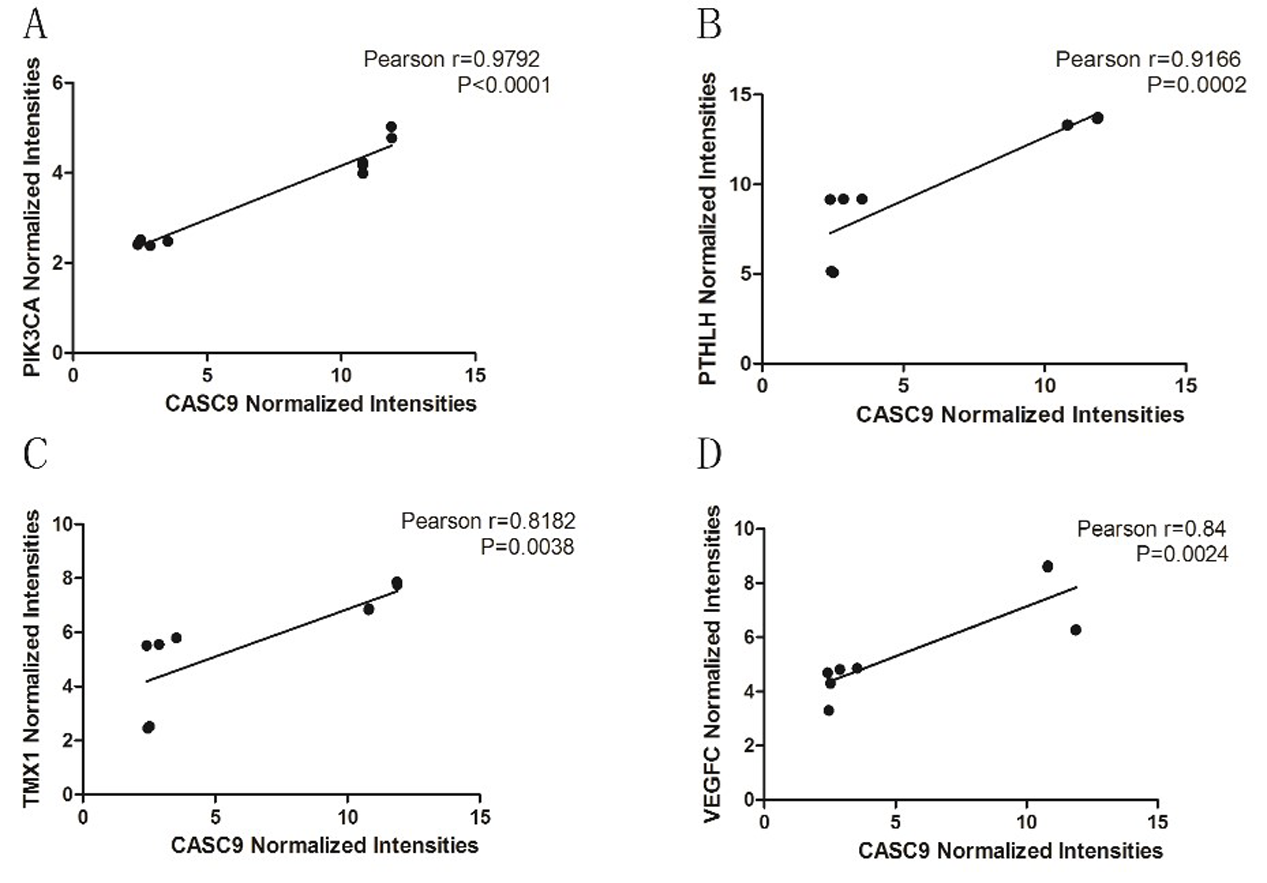


**FigureS4.** Correlation analysis of CASC9 intensities and candidate genes intensities provided by the ESCC tissue profiles. CASC9 intensities positively associates with PIK3CA intensities (A), PTHLH intensities (B), TMX1 intensities (C) and VEGFC intensities (D) respectively.


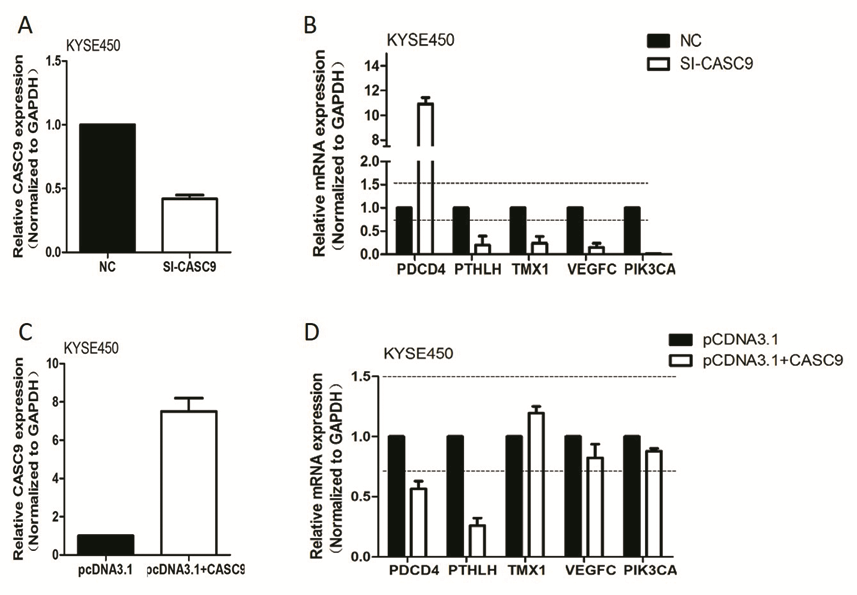


**FigureS5.** qRT-PCR was used to detect the mRNA expression of target genes after interfering and overexpressing CASC9.


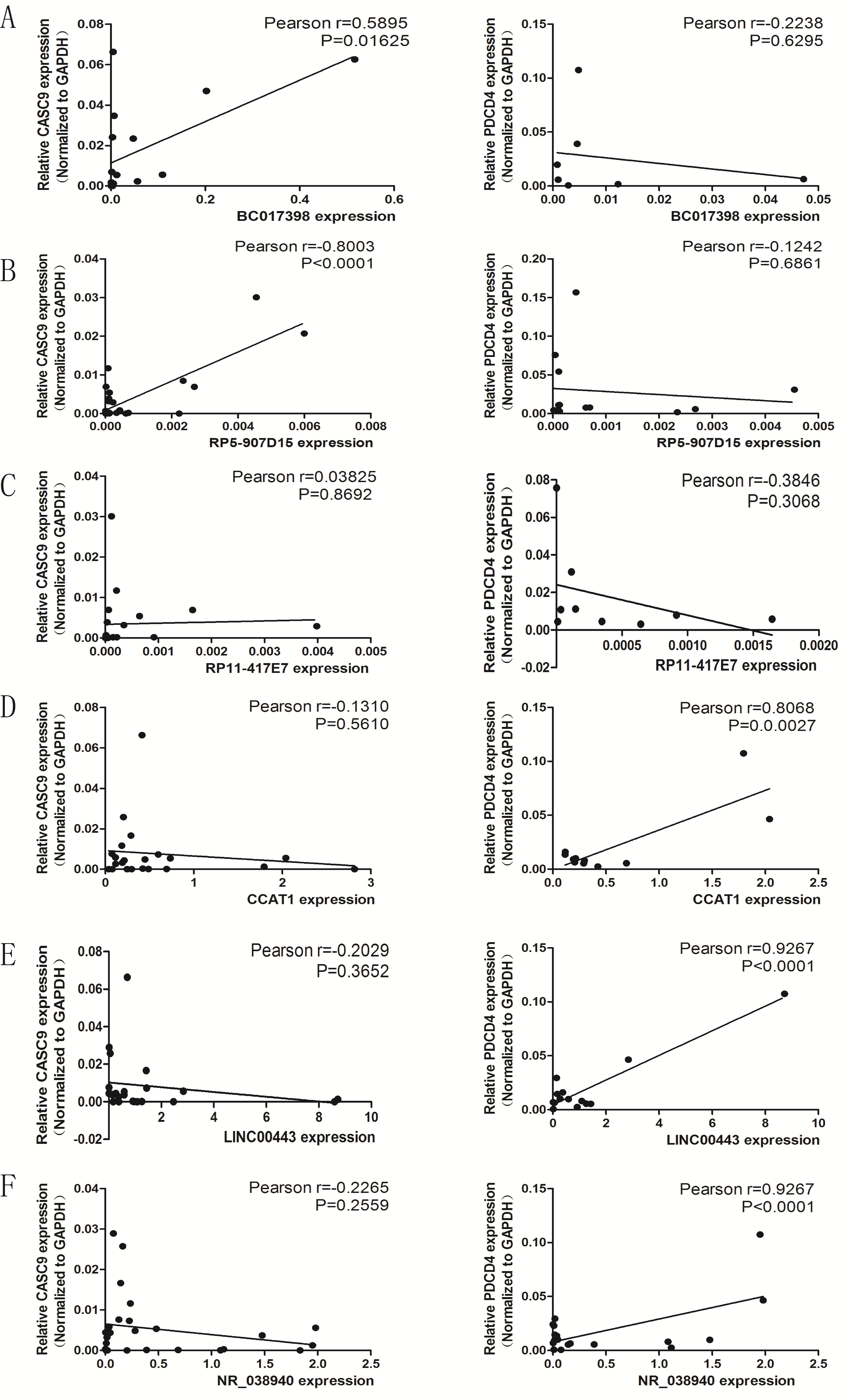


**FigureS6.** Correlation between transcripts identified in Fig.1 and CASC9 or PDCD4. None of them correlates with CASC9 and PDCD4 at the same time.


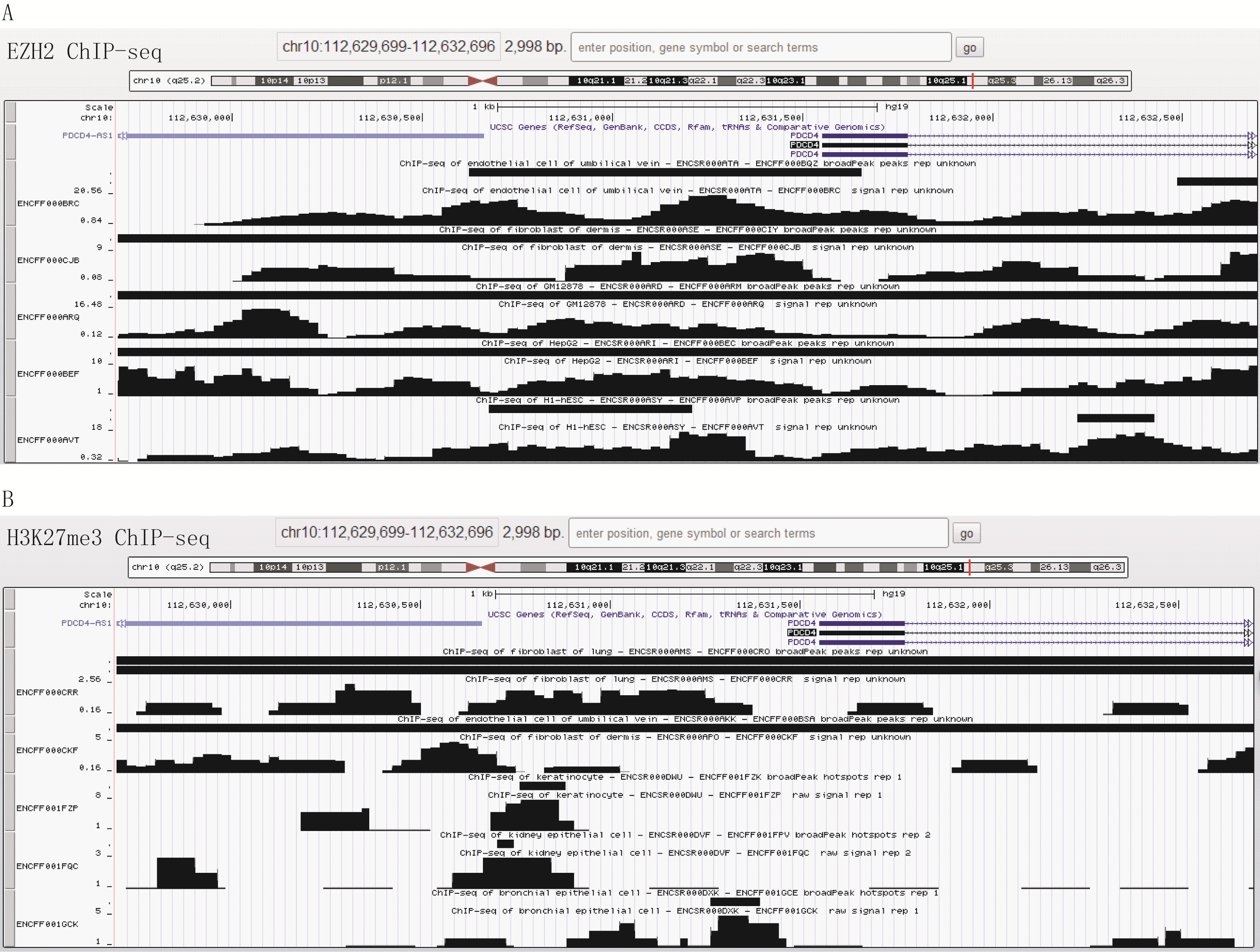


**FigureS7.** ChIP-seq data from Encode indicate that the promoter region of PDCD4 is enriched in the H3K27me3 and EZH2 binding sites.


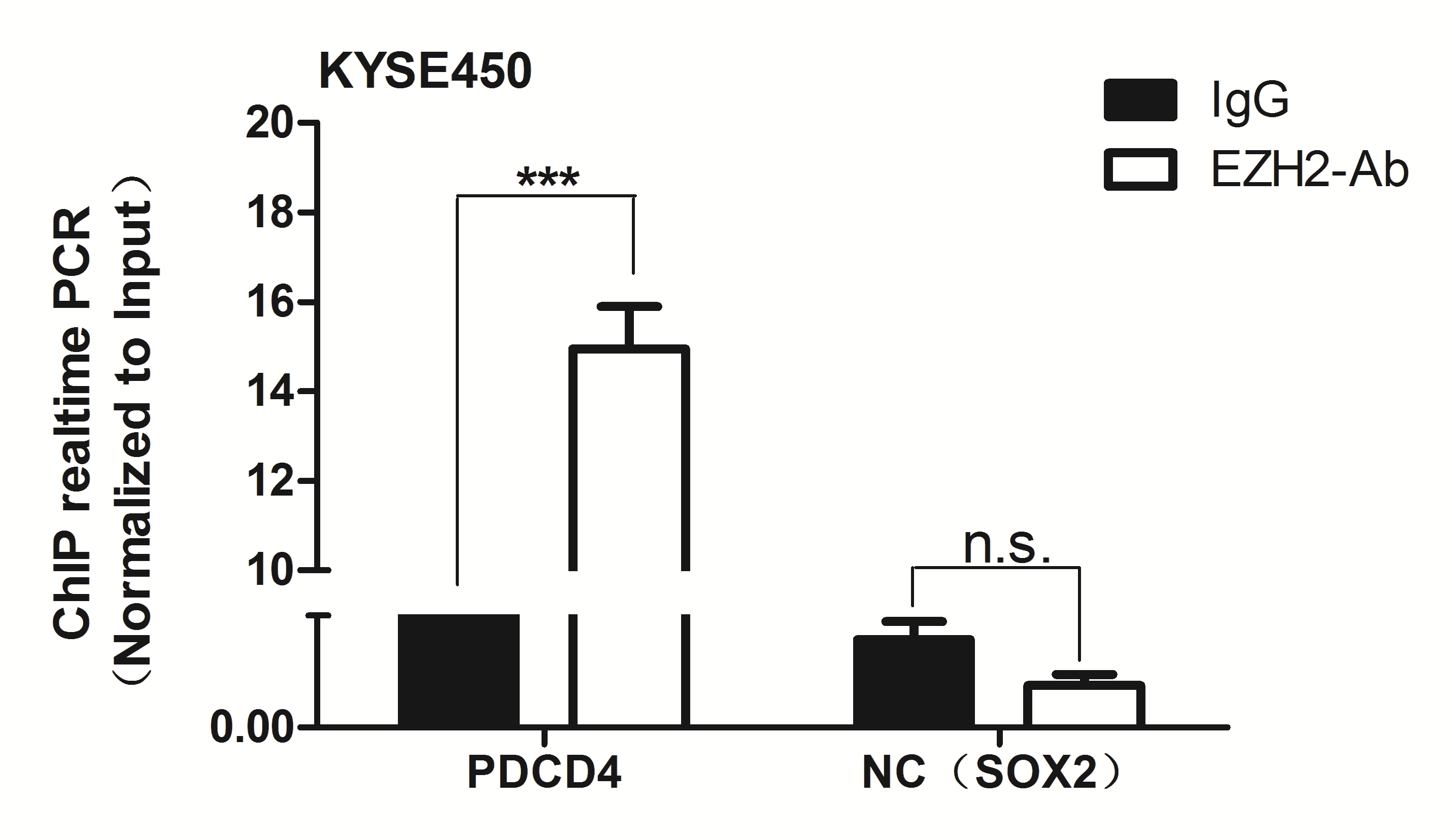


**FigureS8.** ChIP assay showed that EHZ2 could bind to the region of PDCD promoter. SOX2 was used as a negative control. ***P<0.001; n.s, negative significance.


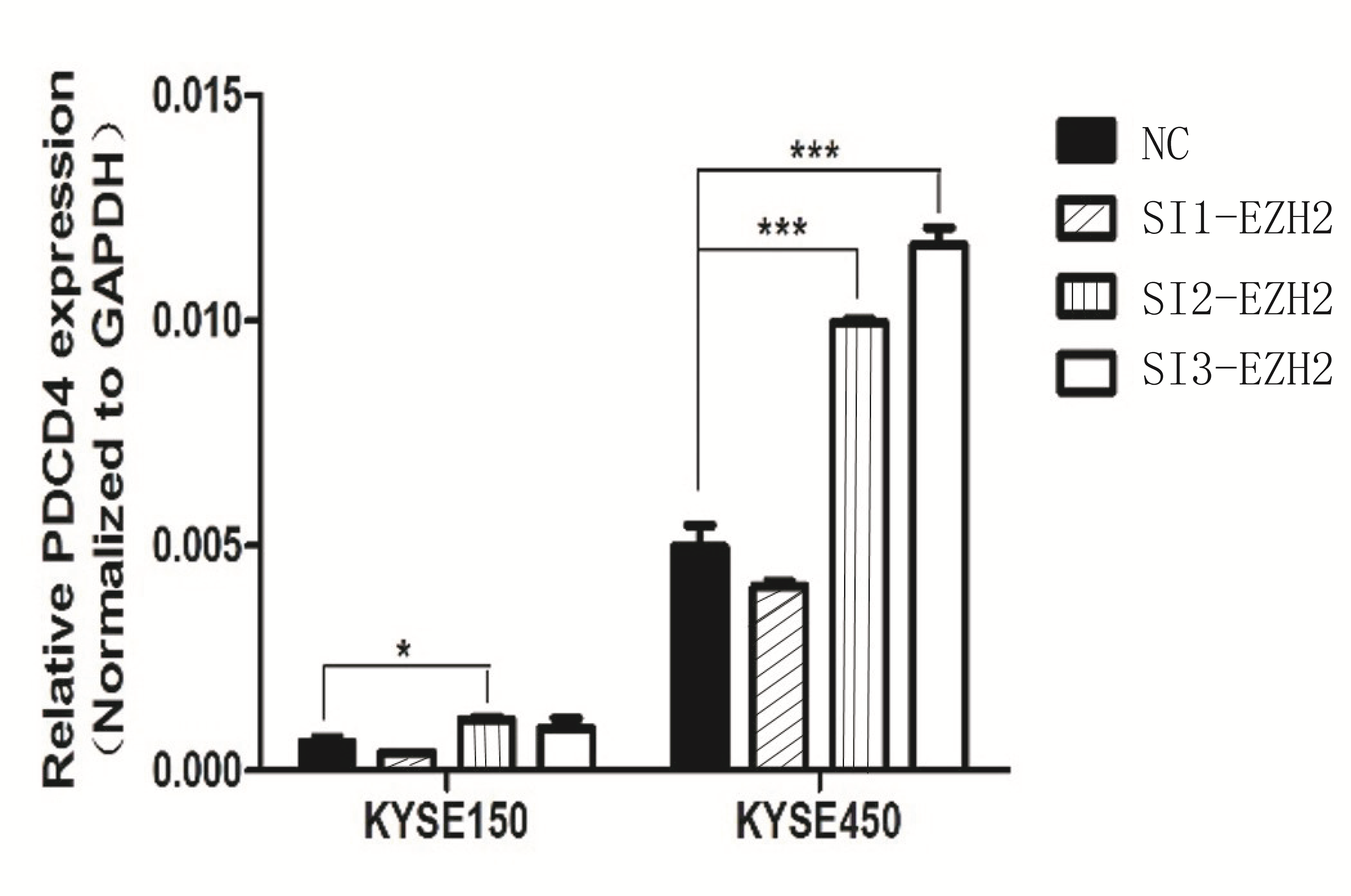


**FigureS9.** PDCD4 mRNA expression after interfering EZH2. Knockdown of EZH2 elevated PDCD4 mRNA expression. *P<0.05, ***P<0.001.
